# Benzyl *N*-[(*S*)-2-hy­droxy-1-({[(*E*)-2-hy­droxy-4-meth­oxy­benzyl­idene]hydrazin­yl}carbon­yl)eth­yl]carbamate

**DOI:** 10.1107/S1600536810047720

**Published:** 2010-11-20

**Authors:** Marcus V. N. de Souza, Alessandra C. Pinheiro, Edward R. T. Tiekink, Solange M. S. V. Wardell, James L. Wardell

**Affiliations:** a Fundação Oswaldo Cruz, Instituto de Tecnologia, em Fármacos – Farmanguinhos, R. Sizenando Nabuco, 100, Manguinhos, 21041-250, Rio de Janeiro, RJ, Brazil; b Department of Chemistry, University of Malaya, 50603 Kuala Lumpur, Malaysia; c CHEMSOL, 1 Harcourt Road, Aberdeen AB15 5NY, Scotland; d Centro de Desenvolvimento Tecnológico em Saúde (CDTS), Fundação Oswaldo Cruz (FIOCRUZ), Casa Amarela, Campus de Manguinhos, Av. Brasil 4365, 21040-900 Rio de Janeiro, RJ, Brazil

## Abstract

The shape of the title compound, C_19_H_21_N_3_O_6_, is curved with the conformation about the imine bond [1.291 (3) Å] being *E*. While the hy­droxy-substituted benzene ring is almost coplanar with the hydrazinyl residue [N—N—C—C = 177.31 (18)°], an observation correlated with an intra­molecular O—H⋯N hydrogen bond leading to an *S*(6) ring, the remaining residues exhibit significant twists. The carbonyl residues are directed away from each other as are the amines. This allows for the formation of O—H⋯O and N—H⋯O hydrogen bonds in the crystal, which lead to two-dimensional supra­molecular arrays in the *ac* plane. Additional stabilization to the layers is afforded by C—H⋯π inter­actions.

## Related literature

For the use of l-serine derivatives in anti-tumour therapy, see: Jiao *et al.* (2009 ▶); Yakura *et al.* (2007 ▶); Takahashi *et al.* (1988 ▶); Sin *et al.* (1998 ▶). For the use of *N*-acyl­hydrazones derivatives from l-serine in anti-tumour testing, see: Rollas & Küçükgüzel (2007 ▶); Terzioğlu & Gürsoy (2003 ▶). For a related structure, see: Pinheiro *et al.* (2010 ▶).
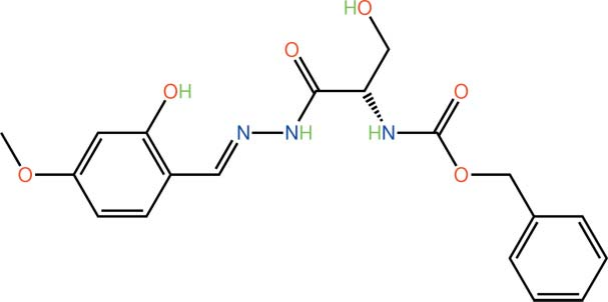


## Experimental

### 

#### Crystal data

C_19_H_21_N_3_O_6_
*M* *_r_* = 387.39
Monoclinic, 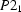

*a* = 5.1634 (2) Å
*b* = 32.3173 (11) Å
*c* = 5.7030 (2) Å
β = 103.918 (2)°
*V* = 923.70 (6) Å^3^
*Z* = 2
Mo *K*α radiation
μ = 0.11 mm^−1^
*T* = 120 K
0.50 × 0.32 × 0.10 mm

#### Data collection

Bruker–Nonius Roper CCD camera on κ-goniostat diffractometer
Absorption correction: multi-scan (*SADABS*; Sheldrick, 2007 ▶) *T*_min_ = 0.623, *T*_max_ = 0.746
9676 measured reflections
2151 independent reflections
1954 reflections with *I* > 2σ(*I*)
*R*_int_ = 0.035

#### Refinement

*R*[*F* ^2^ > 2σ(*F* ^2^)] = 0.032
*wR*(*F* ^2^) = 0.078
*S* = 1.01
2149 reflections
266 parameters
5 restraints
H atoms treated by a mixture of independent and constrained refinement
Δρ_max_ = 0.14 e Å^−3^
Δρ_min_ = −0.19 e Å^−3^

### 

Data collection: *COLLECT* (Hooft, 1998 ▶); cell refinement: *DENZO* (Otwinowski & Minor, 1997 ▶) and *COLLECT*; data reduction: *DENZO* and *COLLECT*; program(s) used to solve structure: *SHELXS97* (Sheldrick, 2008 ▶); program(s) used to refine structure: *SHELXL97* (Sheldrick, 2008 ▶); molecular graphics: *ORTEP-3* (Farrugia, 1997 ▶) and *DIAMOND* (Brandenburg, 2006 ▶); software used to prepare material for publication: *publCIF* (Westrip, 2010 ▶).

## Supplementary Material

Crystal structure: contains datablocks global, I. DOI: 10.1107/S1600536810047720/hb5744sup1.cif

Structure factors: contains datablocks I. DOI: 10.1107/S1600536810047720/hb5744Isup2.hkl

Additional supplementary materials:  crystallographic information; 3D view; checkCIF report

## Figures and Tables

**Table 1:** Hydrogen-bond geometry (Å, °) *Cg*1 is the centroid of the C1–C6 ring.

*D*—H⋯*A*	*D*—H	H⋯*A*	*D*⋯*A*	*D*—H⋯*A*
O1—H1o⋯N1	0.85 (2)	1.91 (2)	2.667 (3)	149 (3)
N2—H2n⋯O4^i^	0.86 (2)	1.89 (2)	2.742 (2)	170 (2)
N3—H3n⋯O5^ii^	0.86 (2)	2.18 (2)	3.013 (2)	165 (2)
O4—H4o⋯O3^iii^	0.84 (2)	1.77 (2)	2.594 (2)	169 (3)
C7—H7c⋯*Cg*1^ii^	0.98	2.67	3.565 (3)	151

Symmetry codes: (i) 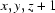; (ii) 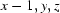; (iii) 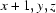.

**Fig. 1.**
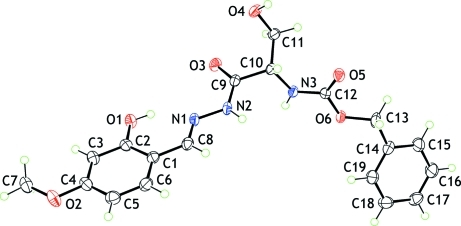
The molecular structure of (I) showing displacement ellipsoids at the 50% probability level.

**Fig. 2.**
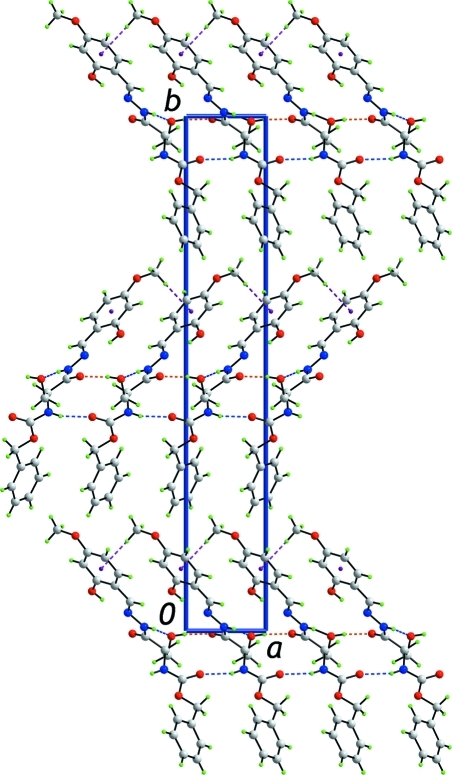
A view in projection down the c axis of the stacking of 2-D supramolecular arrays in the ac plane in (I) with the O—H···O and N—H···O hydrogen bonding shown as orange and blue dashed lines, respectively. The C—H···π interactions are shown as purple dashed lines.

## supplementary crystallographic information

### Comment

Several *L*-serine derivatives have been found to have potential in anti-tumour therapy, for example, conagenin, a naturally occurring serine derivative, was shown to improve the anti-tumour efficacy of adriamycin and mitomycin C against murine leukemias (Jiao *et al.*, 2009; Yakura *et al.*, 2007). Other *L*-serine derivatives reported as potential new anti-tumour agents include the anti-biotic thrazarine, which sensitizes tumour cells to macrophage-mediated cytolysis (Takahashi *et al.*, 1988), and eponemycin, an immunomodulator, which plays a crucial role in tumour progression and metastases by supplying essential nutrients to B16 melanoma cells (Sin *et al.*, 1998).

Following on from such reports, we have synthesized some *N*-acylhydrazones derivatives from *L*-serine to use in anti-tumour testing. The choice of *N*-acylhydrazonyl derivatives was suggested by publications indicating that compounds with such groups can aid anti-tumour activities (Rollas & Küçükgüzel, 2007; Terzioğlu & Gürsoy, 2003). We recently reported the structure of benzyl (1*S*)-2-[2-(2-methoxybenzylidene)hydrazino]-1-(hydroxymethyl)-2-oxoethylcarbamate (Pinheiro *et al.*, 2010) and now we wish to report the structure of the 2-hydroxy-4-methoxy analogue, (I).

Although the absolute structure of (I), Fig. 1, could not be determined experimentally, the assignment of the *S*-configuration at the C10 atom is based on a starting reagent. Overall, the molecule of (I) is curved. The 2-hydroxy-4-methoxyphenyl group is planar [the C7—O2—C4—C3 torsion angle is -1.2 (3) °] and the planarity extends to include the hydrazino residue [N2—N1—C8—C1 = 177.31 (18) °]; the conformation about the N1═C8 bond [1.291 (3) Å] is *E*. The observed planar conformation is stabilized by an intramolecular O1—H···N1 bond that closes an *S*(6) ring, Table 1. A noticeable twist is evident about the hydrazino bond [C8—N1—N2—C9 = -167.97 (19) °], and the remainder of the molecule is similarly twisted. The twists in the molecule results in a conformation where the two carbonyl atoms are directed away from each other, and a similar situation pertains for the amine groups. This arrangement optimizes the formation of a number of strong hydrogen bonds.

In the crystal packing, the O4-hydroxyl group forms an O—H···O hydrogen bond with the carbonyl-O3 atom adjacent to the hydrazino residue, Table 1. Each of the N—H atoms form a N—H···O hydrogen bond: N2 to the O4-hydroxyl group and N3 to the O5-ester atom, Table 1. This results in the formation of supramolecular layers in the *ac* plane, Fig. 2. Additional stabilization to the layers is afforded by C—H···π interactions, Table 1 and Fig. 2.

### Experimental

The compound, phenyl (1*S*)-2-hydrazino-1-(hydroxymethyl)-2-oxoethylcarbamate, was obtained from *L*-serine methyl ester hydrochloride on successive reactions with (*a*) PhCH_2_Cl and Et_3_N, and (*b*) N_2_H_4_.H_2_O. To a stirred ethanol solution (10 ml) of phenyl (1*S*)-2-hydrazino-1-(hydroxymethyl)-2-oxoethylcarbamate (1.0 mmol) at room temperature was added 2-hydroxy-4-methoxybenzaldehyde (1.05 mmol). The reaction mixture was stirred for 4 h at 353 K and concentrated under reduced pressure. The residue was purified by washing with cold ethanol (3 *x* 10 ml), affording (I), yield 80%; m.p. 435 K. The NMR spectra in DMSO solution revealed the presence of (*E*)- and (*Z*)-isomers. However, the colourless needles obtained from methanol were solely the (*E*)-isomer.

^1^H NMR (500 MHz, DMSO-d6) δ (p.p.m.): 11.68 & 11.27 (1*H*, s, NHN, *E* & *Z* isomers), 10.45 & 10.17 (1*H*, s C1—OH, *E* & *Z* isomers), 8.36 & 8.19 (1*H*, s, N═CH, *E* & *Z* isomers), 7.60 (d, J = 8.8 Hz) & 7.47 (d, J = 7.8 Hz), (1*H*, H5, *E* & *Z* isomers), 7.40 (d, J = 8.4 Hz) & 7.38–7.25 (*m*), (1*H*, NHCH, *E* & *Z* isomers), 7.38–7.25 (5*H*, m, Ph), 6.55–6.40 (2*H*, m, H2 & H4), 5.04 (2*H*, s, CH_2_Ph), 5.10–5.00 (*m*) & 4.89 (t, J = 5.9 Hz), (1*H*, OH, *E* & *Z* isomers), 4.93 &.5.11 (1*H*, m, CH, *E* & *Z* isomers), 3,76 & 3.74 (3*H*, s, CH_3_, *E* & *Z* isomers), 3.70–3.55 (2*H*, m, CH_2_OH). ^13^C NMR (125 MHz, DMSO-d6) δ (p.p.m.): 171.2 & 167.0 (COCH, *E* & *Z* isomers), 162.5 & 162.2 (C3, *E* & *Z* isomers), 159.7 & 158.3 (C1, *E* & *Z* isomers) 156.4 (COO), 148.4 & 141.7 (N═CH, *E* & *Z* isomers), 137.4 (C6') 131.5, 128.8, 128.7, 128.3, 128.2 & 127.6 (C5, C1', C2', C3', C4' & C5'), 112.1 (C6), 106.9 (C4), 101.6 (C2), 66.0 & 65.8 (CH_2_Ph, *E* & *Z* isomers), 61.9 & 61.5 (CH_2_OH, *E* & *Z* isomers), 56.7 & 54.9 (CH, *E* & *Z* isomers), 55.8 & 55.6 (CH_3_; *E* & *Z* isomers). IR (cm^-1^; KBr): 3329 (O—H), 1678 (CO). EM/ESI: [M—H]: 386.3.

### Refinement

The C-bound H atoms were geometrically placed (C–H = 0.95–0.99 Å) and refined as riding with *U*_iso_(H) = 1.2–1.5*U*_eq_(C). The O– and N-bound H atoms were located from a difference map and refined with the distance restraint O–H = 0.84 ± 0.01 and N–H = 0.86±0.01 Å, and with *U*_iso_(H) = *zU*_eq_(carrier atom); *z* = 1.5 for O and *z* = 1.2 for N. In the absence of significant anomalous scattering effects, 1934 Friedel pairs were averaged in the final refinement. However, the absolute configuration was assigned on the basis of the chirality of the *L*-serine starting material. In the final refinement two reflections exhibiting poor agreement were omitted, *i.e*. (001) and (011).

### Crystal data

**Table:**

C_19_H_21_N_3_O_6_	*F*(000) = 408
*M**_r_* = 387.39	*D*_x_ = 1.393 Mg m^−^^3^
Monoclinic, *P*2_1_	Mo *K*α radiation, λ = 0.71073 Å
Hall symbol: P 2yb	Cell parameters from 9310 reflections
*a* = 5.1634 (2) Å	θ = 2.9–27.5°
*b* = 32.3173 (11) Å	µ = 0.11 mm^−^^1^
*c* = 5.7030 (2) Å	*T* = 120 K
β = 103.918 (2)°	Prism, colourless
*V* = 923.70 (6) Å^3^	0.50 × 0.32 × 0.10 mm
*Z* = 2	

### Data collection

**Table:**

Bruker–Nonius Roper CCD camera on κ-goniostat diffractometer	2151 independent reflections
Radiation source: Bruker-Nonius FR591 rotating anode	1954 reflections with *I* > 2σ(*I*)
graphite	*R*_int_ = 0.035
Detector resolution: 9.091 pixels mm^-1^	θ_max_ = 27.5°, θ_min_ = 3.7°
φ and ω scans	*h* = −6→6
Absorption correction: multi-scan (*SADABS*; Sheldrick, 2007)	*k* = −41→41
*T*_min_ = 0.623, *T*_max_ = 0.746	*l* = −7→7
9676 measured reflections	

### Refinement

**Table:**

Refinement on *F*^2^	Secondary atom site location: difference Fourier map
Least-squares matrix: full	Hydrogen site location: inferred from neighbouring sites
*R*[*F*^2^ > 2σ(*F*^2^)] = 0.032	H atoms treated by a mixture of independent and constrained refinement
*wR*(*F*^2^) = 0.078	*w* = 1/[σ^2^(*F*_o_^2^) + (0.0441*P*)^2^ + 0.1213*P*] where *P* = (*F*_o_^2^ + 2*F*_c_^2^)/3
*S* = 1.01	(Δ/σ)_max_ = 0.001
2149 reflections	Δρ_max_ = 0.14 e Å^−^^3^
266 parameters	Δρ_min_ = −0.19 e Å^−^^3^
5 restraints	Absolute structure: nd
Primary atom site location: structure-invariant direct methods	

### Special details

**Table:**

Geometry. All s.u.'s (except the s.u. in the dihedral angle between two l.s. planes) are estimated using the full covariance matrix. The cell s.u.'s are taken into account individually in the estimation of s.u.'s in distances, angles and torsion angles; correlations between s.u.'s in cell parameters are only used when they are defined by crystal symmetry. An approximate (isotropic) treatment of cell s.u.'s is used for estimating s.u.'s involving l.s. planes.
Refinement. Refinement of *F*^2^ against ALL reflections. The weighted *R*-factor *wR* and goodness of fit *S* are based on *F*^2^, conventional *R*-factors *R* are based on *F*, with *F* set to zero for negative *F*^2^. The threshold expression of *F*^2^ > 2σ(*F*^2^) is used only for calculating *R*-factors(gt) *etc*. and is not relevant to the choice of reflections for refinement. *R*-factors based on *F*^2^ are statistically about twice as large as those based on *F*, and *R*- factors based on ALL data will be even larger.

### Fractional atomic coordinates and isotropic or equivalent isotropic displacement parameters (Å^2^)

**Table:**

	*x*	*y*	*z*	*U*_iso_*/*U*_eq_	
O1	−0.1786 (3)	1.07693 (5)	0.1104 (3)	0.0267 (3)	
H1O	−0.050 (4)	1.0606 (8)	0.111 (5)	0.040*	
O2	−0.3856 (3)	1.18463 (5)	0.6089 (3)	0.0345 (4)	
O3	0.3291 (3)	0.99440 (5)	−0.1161 (3)	0.0296 (4)	
O4	0.8158 (3)	0.98932 (5)	−0.2785 (3)	0.0292 (3)	
H4O	0.981 (2)	0.9933 (11)	−0.240 (5)	0.044*	
O5	1.1738 (3)	0.91538 (5)	0.3209 (3)	0.0280 (3)	
O6	0.9066 (3)	0.87370 (4)	0.4806 (3)	0.0232 (3)	
N1	0.2721 (4)	1.03527 (5)	0.2864 (3)	0.0229 (4)	
N2	0.4763 (3)	1.00743 (5)	0.2848 (3)	0.0225 (4)	
H2N	0.597 (4)	1.0034 (8)	0.415 (3)	0.027*	
N3	0.7233 (3)	0.92467 (5)	0.2328 (3)	0.0214 (4)	
H3N	0.575 (3)	0.9171 (8)	0.263 (4)	0.026*	
C1	0.1100 (4)	1.08823 (6)	0.5067 (4)	0.0234 (4)	
C2	−0.1163 (4)	1.09787 (7)	0.3212 (4)	0.0225 (4)	
C3	−0.2859 (4)	1.13016 (7)	0.3504 (4)	0.0251 (4)	
H3	−0.4368	1.1368	0.2240	0.030*	
C4	−0.2330 (5)	1.15243 (6)	0.5648 (4)	0.0267 (5)	
C5	−0.0123 (5)	1.14263 (8)	0.7516 (4)	0.0310 (5)	
H5	0.0222	1.1578	0.8987	0.037*	
C6	0.1537 (5)	1.11119 (7)	0.7217 (4)	0.0291 (5)	
H6	0.3029	1.1047	0.8500	0.035*	
C7	−0.6104 (5)	1.19656 (7)	0.4213 (5)	0.0332 (5)	
H7A	−0.5516	1.2027	0.2740	0.050*	
H7B	−0.6934	1.2212	0.4717	0.050*	
H7C	−0.7403	1.1739	0.3896	0.050*	
C8	0.3021 (4)	1.05654 (7)	0.4826 (4)	0.0243 (4)	
H8	0.4518	1.0514	0.6132	0.029*	
C9	0.4915 (4)	0.98936 (6)	0.0787 (4)	0.0205 (4)	
C10	0.7381 (4)	0.96234 (6)	0.0944 (4)	0.0194 (4)	
H10	0.8987	0.9784	0.1799	0.023*	
C11	0.7671 (4)	0.95243 (7)	−0.1585 (4)	0.0243 (4)	
H11A	0.9168	0.9329	−0.1487	0.029*	
H11B	0.6019	0.9390	−0.2521	0.029*	
C12	0.9530 (4)	0.90556 (6)	0.3433 (4)	0.0215 (4)	
C13	1.1447 (4)	0.85176 (7)	0.6038 (4)	0.0309 (5)	
H13A	1.2736	0.8713	0.7031	0.037*	
H13B	1.2301	0.8387	0.4844	0.037*	
C14	1.0676 (4)	0.81910 (7)	0.7625 (4)	0.0269 (5)	
C15	1.1859 (5)	0.78017 (7)	0.7770 (5)	0.0355 (6)	
H15	1.3004	0.7736	0.6746	0.043*	
C16	1.1382 (5)	0.75094 (8)	0.9394 (5)	0.0394 (6)	
H16	1.2225	0.7247	0.9493	0.047*	
C17	0.9694 (5)	0.75974 (8)	1.0862 (4)	0.0347 (5)	
H17	0.9395	0.7398	1.1991	0.042*	
C18	0.8429 (5)	0.79800 (8)	1.0684 (4)	0.0358 (6)	
H18	0.7217	0.8039	1.1656	0.043*	
C19	0.8938 (5)	0.82745 (7)	0.9084 (4)	0.0315 (5)	
H19	0.8087	0.8537	0.8984	0.038*	

### Atomic displacement parameters (Å^2^)

**Table:**

	*U*^11^	*U*^22^	*U*^33^	*U*^12^	*U*^13^	*U*^23^
O1	0.0242 (8)	0.0238 (8)	0.0305 (8)	0.0034 (6)	0.0034 (6)	−0.0037 (6)
O2	0.0306 (9)	0.0285 (8)	0.0460 (10)	0.0027 (7)	0.0122 (7)	−0.0117 (7)
O3	0.0182 (7)	0.0414 (9)	0.0264 (8)	0.0038 (7)	−0.0003 (6)	0.0081 (7)
O4	0.0185 (7)	0.0398 (9)	0.0280 (7)	−0.0006 (7)	0.0031 (6)	0.0156 (7)
O5	0.0189 (7)	0.0290 (8)	0.0369 (8)	0.0027 (6)	0.0084 (6)	0.0102 (7)
O6	0.0179 (7)	0.0238 (7)	0.0278 (8)	0.0034 (6)	0.0052 (6)	0.0083 (6)
N1	0.0204 (9)	0.0178 (9)	0.0309 (9)	0.0042 (7)	0.0070 (7)	0.0049 (7)
N2	0.0186 (8)	0.0203 (8)	0.0268 (9)	0.0048 (7)	0.0022 (7)	0.0048 (7)
N3	0.0163 (8)	0.0220 (9)	0.0263 (9)	0.0011 (6)	0.0062 (7)	0.0071 (7)
C1	0.0250 (11)	0.0201 (10)	0.0265 (11)	−0.0013 (8)	0.0088 (8)	0.0037 (8)
C2	0.0219 (10)	0.0199 (10)	0.0278 (11)	−0.0037 (8)	0.0098 (8)	0.0005 (8)
C3	0.0196 (10)	0.0229 (10)	0.0332 (11)	−0.0014 (8)	0.0071 (8)	−0.0020 (9)
C4	0.0281 (11)	0.0193 (10)	0.0368 (13)	−0.0021 (8)	0.0162 (9)	−0.0020 (9)
C5	0.0379 (13)	0.0275 (12)	0.0288 (11)	−0.0028 (10)	0.0102 (10)	−0.0021 (9)
C6	0.0336 (12)	0.0276 (11)	0.0248 (11)	0.0013 (10)	0.0043 (9)	0.0028 (9)
C7	0.0298 (12)	0.0246 (11)	0.0480 (14)	0.0019 (9)	0.0147 (10)	−0.0025 (11)
C8	0.0245 (10)	0.0204 (10)	0.0283 (11)	0.0000 (8)	0.0069 (8)	0.0054 (9)
C9	0.0165 (9)	0.0198 (10)	0.0244 (10)	−0.0010 (8)	0.0036 (7)	0.0049 (8)
C10	0.0154 (9)	0.0210 (10)	0.0207 (9)	−0.0016 (8)	0.0020 (7)	0.0046 (8)
C11	0.0245 (11)	0.0261 (11)	0.0225 (10)	0.0002 (8)	0.0059 (8)	0.0040 (9)
C12	0.0213 (10)	0.0213 (10)	0.0224 (9)	0.0024 (8)	0.0065 (8)	0.0027 (8)
C13	0.0222 (11)	0.0295 (12)	0.0396 (13)	0.0071 (9)	0.0047 (9)	0.0159 (11)
C14	0.0243 (11)	0.0252 (11)	0.0286 (11)	−0.0002 (8)	0.0010 (9)	0.0058 (9)
C15	0.0369 (14)	0.0303 (12)	0.0428 (14)	0.0085 (10)	0.0166 (11)	0.0103 (11)
C16	0.0460 (15)	0.0262 (12)	0.0500 (15)	0.0085 (11)	0.0192 (12)	0.0128 (11)
C17	0.0452 (15)	0.0279 (12)	0.0314 (12)	−0.0017 (10)	0.0099 (10)	0.0071 (10)
C18	0.0459 (15)	0.0346 (13)	0.0306 (12)	0.0000 (11)	0.0167 (11)	0.0017 (10)
C19	0.0368 (13)	0.0239 (12)	0.0344 (13)	0.0038 (9)	0.0098 (10)	0.0028 (10)

### Geometric parameters (Å, °)

**Table:**

O1—C2	1.349 (3)	C5—H5	0.9500
O1—H1O	0.846 (10)	C6—H6	0.9500
O2—C4	1.365 (3)	C7—H7A	0.9800
O2—C7	1.430 (3)	C7—H7B	0.9800
O3—C9	1.231 (2)	C7—H7C	0.9800
O4—C11	1.427 (3)	C8—H8	0.9500
O4—H4O	0.840 (10)	C9—C10	1.529 (3)
O5—C12	1.219 (3)	C10—C11	1.519 (3)
O6—C12	1.349 (2)	C10—H10	1.0000
O6—C13	1.446 (2)	C11—H11A	0.9900
N1—C8	1.291 (3)	C11—H11B	0.9900
N1—N2	1.388 (2)	C13—C14	1.505 (3)
N2—C9	1.332 (3)	C13—H13A	0.9900
N2—H2N	0.857 (10)	C13—H13B	0.9900
N3—C12	1.351 (3)	C14—C19	1.389 (3)
N3—C10	1.463 (3)	C14—C15	1.393 (3)
N3—H3N	0.861 (10)	C15—C16	1.385 (4)
C1—C6	1.404 (3)	C15—H15	0.9500
C1—C2	1.410 (3)	C16—C17	1.376 (4)
C1—C8	1.455 (3)	C16—H16	0.9500
C2—C3	1.398 (3)	C17—C18	1.391 (4)
C3—C4	1.388 (3)	C17—H17	0.9500
C3—H3	0.9500	C18—C19	1.387 (3)
C4—C5	1.396 (3)	C18—H18	0.9500
C5—C6	1.366 (3)	C19—H19	0.9500
			
C2—O1—H1O	107 (2)	N2—C9—C10	115.08 (16)
C4—O2—C7	117.93 (18)	N3—C10—C11	111.51 (16)
C11—O4—H4O	107 (2)	N3—C10—C9	110.83 (16)
C12—O6—C13	114.04 (16)	C11—C10—C9	109.62 (16)
C8—N1—N2	114.69 (18)	N3—C10—H10	108.3
C9—N2—N1	119.74 (17)	C11—C10—H10	108.3
C9—N2—H2N	120.9 (19)	C9—C10—H10	108.3
N1—N2—H2N	119.3 (18)	O4—C11—C10	110.37 (17)
C12—N3—C10	118.55 (17)	O4—C11—H11A	109.6
C12—N3—H3N	120.1 (17)	C10—C11—H11A	109.6
C10—N3—H3N	120.6 (18)	O4—C11—H11B	109.6
C6—C1—C2	117.9 (2)	C10—C11—H11B	109.6
C6—C1—C8	119.0 (2)	H11A—C11—H11B	108.1
C2—C1—C8	123.06 (19)	O5—C12—O6	124.06 (19)
O1—C2—C3	117.29 (19)	O5—C12—N3	124.77 (19)
O1—C2—C1	122.32 (19)	O6—C12—N3	111.17 (17)
C3—C2—C1	120.38 (19)	O6—C13—C14	108.60 (18)
C4—C3—C2	119.7 (2)	O6—C13—H13A	110.0
C4—C3—H3	120.2	C14—C13—H13A	110.0
C2—C3—H3	120.2	O6—C13—H13B	110.0
O2—C4—C3	123.9 (2)	C14—C13—H13B	110.0
O2—C4—C5	115.7 (2)	H13A—C13—H13B	108.4
C3—C4—C5	120.4 (2)	C19—C14—C15	118.5 (2)
C6—C5—C4	119.6 (2)	C19—C14—C13	121.8 (2)
C6—C5—H5	120.2	C15—C14—C13	119.6 (2)
C4—C5—H5	120.2	C16—C15—C14	120.6 (2)
C5—C6—C1	121.9 (2)	C16—C15—H15	119.7
C5—C6—H6	119.1	C14—C15—H15	119.7
C1—C6—H6	119.1	C17—C16—C15	120.4 (2)
O2—C7—H7A	109.5	C17—C16—H16	119.8
O2—C7—H7B	109.5	C15—C16—H16	119.8
H7A—C7—H7B	109.5	C16—C17—C18	119.7 (2)
O2—C7—H7C	109.5	C16—C17—H17	120.2
H7A—C7—H7C	109.5	C18—C17—H17	120.2
H7B—C7—H7C	109.5	C19—C18—C17	119.9 (2)
N1—C8—C1	120.96 (19)	C19—C18—H18	120.1
N1—C8—H8	119.5	C17—C18—H18	120.1
C1—C8—H8	119.5	C18—C19—C14	120.9 (2)
O3—C9—N2	124.50 (19)	C18—C19—H19	119.6
O3—C9—C10	120.36 (19)	C14—C19—H19	119.6
			
C8—N1—N2—C9	−167.97 (19)	C12—N3—C10—C9	−154.94 (18)
C6—C1—C2—O1	−178.4 (2)	O3—C9—C10—N3	−112.2 (2)
C8—C1—C2—O1	3.3 (3)	N2—C9—C10—N3	70.4 (2)
C6—C1—C2—C3	1.8 (3)	O3—C9—C10—C11	11.3 (3)
C8—C1—C2—C3	−176.6 (2)	N2—C9—C10—C11	−166.09 (17)
O1—C2—C3—C4	179.26 (19)	N3—C10—C11—O4	−172.47 (16)
C1—C2—C3—C4	−0.9 (3)	C9—C10—C11—O4	64.4 (2)
C7—O2—C4—C3	−1.2 (3)	C13—O6—C12—O5	0.5 (3)
C7—O2—C4—C5	178.6 (2)	C13—O6—C12—N3	179.84 (18)
C2—C3—C4—O2	179.4 (2)	C10—N3—C12—O5	−6.3 (3)
C2—C3—C4—C5	−0.3 (3)	C10—N3—C12—O6	174.38 (16)
O2—C4—C5—C6	−179.1 (2)	C12—O6—C13—C14	176.21 (18)
C3—C4—C5—C6	0.7 (3)	O6—C13—C14—C19	−45.1 (3)
C4—C5—C6—C1	0.3 (4)	O6—C13—C14—C15	139.2 (2)
C2—C1—C6—C5	−1.5 (3)	C19—C14—C15—C16	−2.2 (4)
C8—C1—C6—C5	176.9 (2)	C13—C14—C15—C16	173.6 (2)
N2—N1—C8—C1	177.31 (18)	C14—C15—C16—C17	1.1 (4)
C6—C1—C8—N1	−178.7 (2)	C15—C16—C17—C18	1.0 (4)
C2—C1—C8—N1	−0.4 (3)	C16—C17—C18—C19	−2.0 (4)
N1—N2—C9—O3	−2.6 (3)	C17—C18—C19—C14	0.8 (4)
N1—N2—C9—C10	174.73 (17)	C15—C14—C19—C18	1.2 (4)
C12—N3—C10—C11	82.6 (2)	C13—C14—C19—C18	−174.5 (2)

### Hydrogen-bond geometry (Å, °)

**Table:**

Cg1 is the centroid of the C1–C6 ring.

**Table:**

*D*—H···*A*	*D*—H	H···*A*	*D*···*A*	*D*—H···*A*
O1—H1o···N1	0.85 (2)	1.91 (2)	2.667 (3)	149 (3)
N2—H2n···O4^i^	0.857 (18)	1.894 (18)	2.742 (2)	170 (2)
N3—H3n···O5^ii^	0.860 (18)	2.175 (17)	3.013 (2)	165 (2)
O4—H4o···O3^iii^	0.838 (15)	1.766 (16)	2.594 (2)	169 (3)
C7—H7c···Cg1^ii^	0.98	2.67	3.565 (3)	151

Symmetry codes:  (i) *x*, *y*, *z*+1; (ii) *x*−1, *y*, *z*; (iii) *x*+1, *y*, *z*.
